# Evaluating the Anti-Neuroinflammatory Capacity of Raw and Steamed Garlic as Well as Five Organosulfur Compounds

**DOI:** 10.3390/molecules191117697

**Published:** 2014-10-31

**Authors:** Su-Chen Ho, Min-Sheng Su

**Affiliations:** Department of Food Science, Yuanpei University, No. 306, Yuanpei Street, Hsinchu 300, Taiwan; E-Mail: mssu@mail.ypu.edu.tw

**Keywords:** garlic, organosulfur compounds, diallyl trisulfide, neuroinflammation, microglia

## Abstract

The anti-neuroinflammatory capacities of raw and steamed garlic extracts as well as five organosulfur compounds (OSCs) were examined in lipopolysaccharide (LPS)-stimulated BV2 microglia. According to those results, steaming pretreatment blocked the formation of alliinase-catalyzed OSCs such as allicin and diallyl trisulfide (DATS) in crushed garlic. Raw garlic, but not steamed garlic, dose-dependently attenuated the production of LPS-induced nitric oxide (NO), interleukin-1β (IL-1β), tumor necrosis factor (TNF)-α, and monocyte chemoattractant protein-1 (MCP-1). DATS and diallyl disulfide at 200 and 400 μM, respectively, displayed significant anti-neuroinflammatory activity. Meanwhile, even at 1 mM, diallyl sulfide, S-allyl cysteine and alliin did not display such activity. Inhibition of nuclear factor-κB activation was the mechanism underlying this protective effect of raw garlic and DATS. Analysis results indicated that the anti-neuroinflammatory capacity of raw garlic is due to the alliin-derived OSCs. Importantly, DATS is a highly promising therapeutic candidate for treating inflammation-related neurodegenerative diseases.

## 1. Introduction

Microglial cells are the prime effector cells responsible for immune defense and inflammatory response of the central nervous system [1]. In response to pathogen invasion or tissue damage, microglial cells are activated and secrete various cytotoxic, pro-inflammatory and chemoattractant factors, including nitric oxide (NO), reactive oxygen species, tumor necrosis factor-α (TNF-α), interleukin-1β (IL-1β), eicosanoids and monocyte chemoattractant protein-1 (MCP-1) in order to remove the damaged cells and debris, eventually restoring homeostasis [2,3]. However, long-term uncontrolled microglial activation causes neuroinflammation, and the sustainably secreted cytotoxic and proinflammatory factors act directly on neurons to induce apoptosis [1,4]. Undoubtedly, more than a hallmark, neuroinflammation is also highly involved in the pathogenesis of many neurodegenerative diseases, including Alzheimer’s disease, Parkinson’s disease, amyotrophic lateral sclerosis (ALS) and multiple sclerosis [2,5]. Consequently, inhibition of pro-inflammatory mediators secreted from activated microglia is a highly promising means of exploiting functional foods to treat neurodegenerative diseases [5,6,7].

Garlic (*Allium sativum* L.), belonging to the lily family, has been used for culinary and medicinal purposes since ancient times. As a folk remedy, garlic is commonly used to control diseases such as microbial infections, hyperlipidemia, and heart diseases [8]. Garlic possesses a diverse array of pharmaceutical activities, including anti-cancer, anti-diabetes, anti-atherosclerosis, hepatoprotective and anti-inflammatory effects. As is widely assumed, these health benefits of garlic are largely due to its characterized flavoring constituents, *i.e.*, organosulfur compounds (OSCs) [8,9,10]. In addition to cultivation and storage conditions, the composition of OSCs in different garlic preparations depends mainly on their processing methods. More than the most abundant naturally occurring OSCs in raw garlic, γ-glutamyl-S-alk(en)yl-l-cysteine and S-allyl-l-cysteine sulfoxide (alliin) are also the parent compounds of OSCs in different garlic preparations. Alliin, which accounts for approximately 80% of the cysteine sulfoxides in raw garlic, is derived from the oxidation of γ-glutamyl-S-alk(en)yl-l-cysteine during the early developmental stage of garlic plant. When raw garlic is crushed, cut, and chewed, alliinase is released and alliin is then converted into diallyl thiosulfinate (allicin). However, allicin is unstable and readily decomposed into oil-soluble OSCs (e.g., diallyl sulfide (DAS), diallyl disulfide (DADS), diallyl trisulfide (DATS), vinyldithiins, and ajoene) and some water-soluble OSCs (e.g., S-allyl cysteine (SAC) and S-allyl mercaptocysteine). Consequently, these odorous oil-soluble OSCs, especially DAS, DADS, and DATS, are the principal compounds of distilled garlic oil. In contrast, aged garlic extract is rich in odorless water-soluble SAC and S-allylmercaptocysteine [10,11].

Capable of inhibiting NO and inflammatory cytokine production in LPS-treated macrophages, multiple garlic preparations and OSCs exhibit an anti-inflammatory capacity [12,13,14,15]. Additionally, heating causes alliinase inactivation, thereby blocking subsequent odorous OSCs formation, which is assumed to be related to the reduction of garlic’s bioactivity, such as its anti-carcinogenic and anti-inflammatory properties [15,16]. Nevertheless, whether garlic can inhibit activated microglia-mediated neuroinflammation and whether heating can eliminate the anti-neuroinflammatory capacity of garlic remain unclear. Therefore, this study first evaluates the anti-neuroinflammatory capacities and, simultaneously, analyzes and compares the OSC compositions of raw and steamed garlic extracts by LC-MS. Moreover, five OSCs are selected to evaluate their anti-neuroinflammatory capacities in order to more thoroughly elucidate the structure-activity relationship. Additionally, the anti-neuroinflammatory capacities of garlic extracts and OSCs are evaluated, owing to their ability to inhibit the secretion and expression of NO, TNF-α, IL-1β, and MCP-1, as well as the DNA binding activity of the upstream convergent transcription factor, nuclear factor-κB (NF-κB), in LPS-activated BV2 microglial cells.

## 2. Results and Discussion

### 2.1. Organosulfur Compositional Changes after Steaming

**Figure 1:**
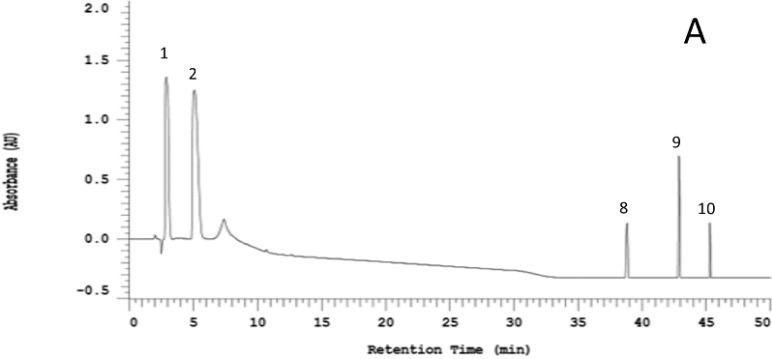

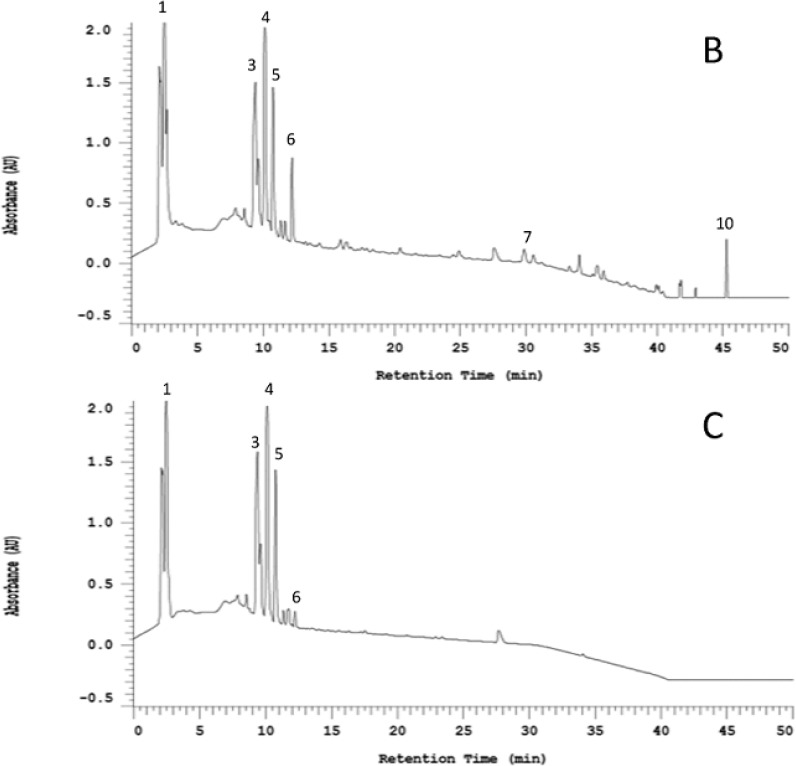
Representative HPLC chromatogram of selected organosulfur standards (**A**); raw garlic extract (**B**); and steamed garlic extract (**C**). Peaks: (1) alliin, (2) SAC, (3) γ-glutamyl-S-allyl-l-cysteine, (4) γ-glutamyl-S-(trans-1-propenyl)-l-cysteine, (5) γ-glutamyl phenylalanine, (6) S-allyl mercaptocysteine, (7) allicin, (8) DAS, (9) DADS, and (10) DATS.

Figure 1 shows a representative HPLC chromatogram for the five OSCs as well as the raw and steamed garlic ethanolic extracts. According to the HPLC chromatogram, the alliin content of raw garlic was obviously as abundant as that of steamed garlic. This finding suggests that 10 min of incubation after crushing was insufficient for alliinase to totally catalyze alliin degradation. Neither raw garlic nor steamed garlic contained SAC. Moreover, raw garlic extract contained several OSCs which were not found in the steamed garlic extract; these distinguishable OSCs were more oil-soluble. Apparently, DATS was more significantly abundant in raw garlic than that in steamed garlic. Additionally, some peak compounds in garlic extract without corresponding standards were verified by comparing mass spectrum with previous report [17]. Peaks 1 through 7 were identified as follows: alliin (MW 177) *m*/*z* = 178 [M+H]^+^, SAC (MW 161) *m*/*z* = 162 [M+H]^+^, γ-glutamyl-S-allyl-l-cysteine (MW 290) *m*/*z* = 291 [M+H]^+^, γ-glutamyl-S-(trans-1-propenyl)-l-cysteine (MW 290) *m*/*z* = 291 [M+H]^+^, γ-glutamyl phenylalanine (MW 294) *m*/*z* = 295 [M+H]^+^, S-allyl mercaptocysteine (MW 193) *m*/*z* = 194 [M+H]^+^ and allicin (MW 162) *m*/*z* = 163 [M+H]^+^, respectively. Interestingly, the S-allyl- mercaptocysteine content of raw garlic was significantly higher than that of steamed garlic. Allicin only appeared in the raw garlic extract. Above results indicate that steaming pretreatment resulted in alliinase inactivation, subsequently blocking the formation of alliin-derived OSCs, such as DATS and allicin.

### 2.2. Anti-Neuroinflammatory Activities of Raw and Steamed Garlic Extracts

#### 2.2.1. Inhibitory Effect of Raw and Steamed Garlic Extracts on NO Production and iNOS Gene Expression

**Figure 2:**
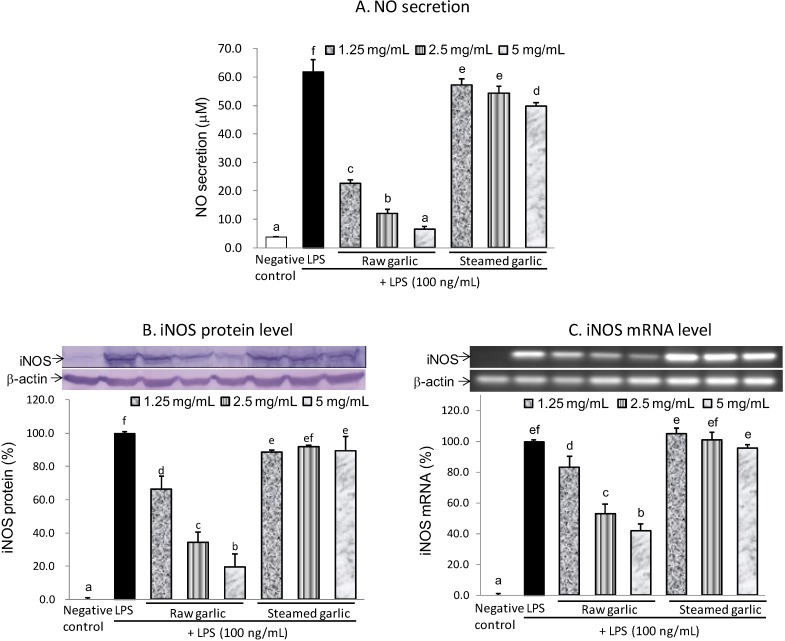
Effects of raw and steamed garlic extracts on the NO production (**A**); iNOS protein (**B**) and iNOS mRNA (**C**) levels in LPS-activated BV2 microglia. The treatment concentrations of garlic extract are 1.25–5 mg/mL. The values are expressed as means ± SD of triplicate tests. Means not sharing a common letter are significantly different (*p* < 0.05) when analyzed by ANOVA and Duncan’s multiple range test.

Given that raw and steamed garlic ethanolic extracts did not cause cytotoxicity for BV2 cells at a concentration of 5 mg/mL, the anti-neuroinflammatory capacity of garlic extract was evaluated at concentrations of ≤5 mg/mL. According to Figure 2A, both the raw and steamed garlic extracts diminished LPS-induced NO production in a dose-dependent manner at concentrations of 1.25–5 mg/mL. The inhibitory capacity of raw garlic extract on LPS-induced NO production was more potent than that of the steamed garlic extract. At 5 mg/mL, raw garlic extract nearly inhibited LPS-induced NO production completely (95.2%), while steamed garlic extract only inhibited 20.6% (Table 1). Furthermore, the extent to which these two garlic extracts inhibit the expression of NO producing enzyme was examined by determining the protein and mRNA levels of iNOS, by using immunoblotting and RT-PCR analysis, respectively. According to Figure 2B,C and Table 1, raw garlic extract dose-dependently attenuated both the iNOS protein and mRNA expression at concentrations of 1.25–5 mg/mL, while steamed garlic extract did not. At 5 mg/mL, raw garlic extract attenuated 80.7% and 58.2%, respectively, of LPS-induced iNOS protein and mRNA expression. Apparently, raw garlic extract had a potent inhibitory capacity on LPS-induced NO production mainly through the down-regulation of iNOS gene expression. Furthermore, steaming treatment diminished this inhibitory capacity.

**Table 1:** Inhibitory rate of raw and steamed garlic extracts on the proinflammatory mediator secretion and mRNA expression in LPS-activated RAW 264.7 macrophages.

	Negative Control	LPS	Raw Garlic			Steamed Garlic		
			1.25 mg/mL	2.5 mg/mL	5.0 mg/mL	1.25 mg/mL	2.5 mg/mL	5.0 mg/mL
Inhibition of NO secretion (%)	100.0	0.0	67.6	85.5	95.2	8.0	12.7	20.6
Inhibition of iNOS protein (%)	100.0	0.0	33.8	65.8	80.7	11.5	8.3	10.8
Inhibition of iNOS mRNA (%)	100.0	0.0	16.9	47.3	58.2	−5.1	−1.1	4.3
Inhibition of TNF−α secretion (%)	100.0	0.0	10.5	36.9	66.0	−2.2	−0.6	9.1
Inhibition of IL−1β secretion (%)	100.0	0.0	14.2	31.9	49.1	−5.4	7.1	8.5
Inhibition of MCP−1 secretion (%)	100.0	0.0	45.8	73.8	94.6	4.2	5.7	7.9
Inhibition of TNF−α mRNA (%)	100.0	0.0	4.3	18.8	62.6	−2.8	−2.8	−1.0
Inhibition of IL−1β mRNA (%)	100.0	0.0	−4.3	17.1	34.1	2.9	6.3	14.2
Inhibition of MCP−1 mRNA (%)	100.0	0.0	29.8	57.4	72.3	−4.1	−1.3	12.6

**Figure 3:**
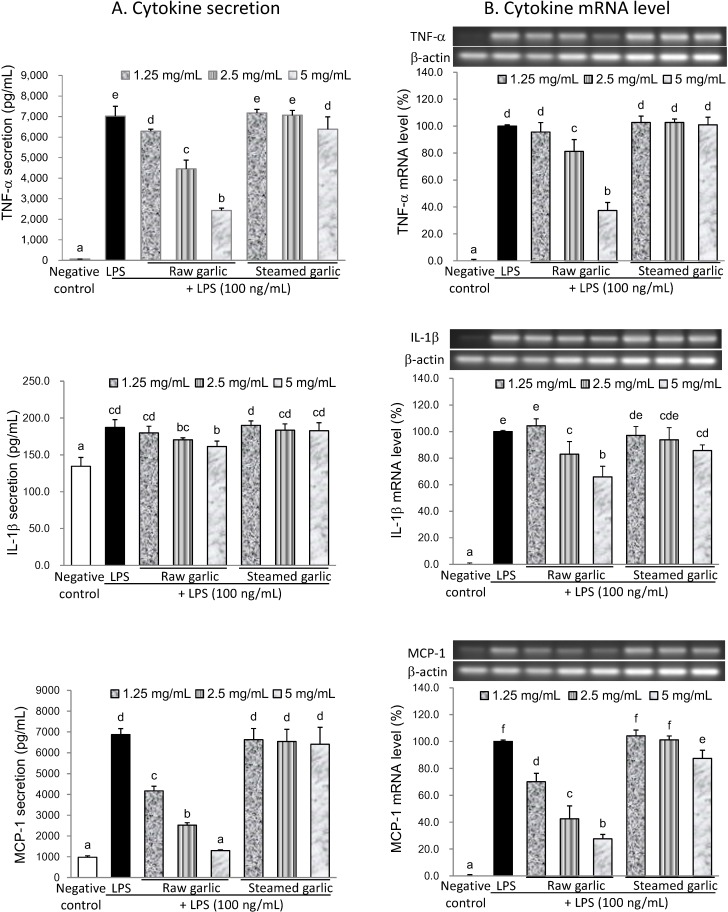
Effects of raw and steamed garlic extracts on TNF-α, IL-1β and MCP-1 secretion (**A**) and mRNA level (**B**) in LPS-activated BV2 microglia. The treatment concentrations of garlic extract are 1.25–5.00 mg/mL. The values are expressed as means ± SD of triplicate tests. Means not sharing a common letter are significantly different (*p* < 0.05) when analyzed by ANOVA and Duncan’s multiple range test.

#### 2.2.2. Inhibitory Effect of Raw and Steamed Garlic Extracts on Proinflammatory TNF-α, IL-1β, and MCP-1 Secretion and Gene Expression

According to Figure 3 and Table 1, raw garlic extract significantly inhibited LPS-induced secretion and expression of proinflammatory cytokines, while steamed garlic extract did not inhibit them. Raw garlic extract at a concentration of 5 mg/mL diminished TNF-α, IL-1β, and MCP-1 secretion in LPS-activated BV2 microglia by 66.0%, 49.1%, and 94.6%, respectively. Simultaneously, raw garlic extract reduced the mRNA level of TNF-α, IL-1β, and MCP-1 by 62.6%, 34.1%, and 72.3%, respectively. Correspondingly, raw garlic extract suppressed inflammatory cytokine secretion mainly through down-regulation of these inflammatory gene expressions. The steaming process diminished this inhibitory capacity. Above results suggest the reduction of anti-neuroinflammatory capacity of garlic, owing to that steaming was related to the inactivation of alliinase and subsequent loss of alliin-derived OSCs formation, especially those of oil-soluble OSCs such as allicin and DATS. Shin *et al.* made a similar observation, indicating that short-term heating treatment (95 °C for 2 h) during extraction decreased the anti-inflammatory activity of crushed raw garlic, and was closely related to the reduced allicin level [15]. Another study found that, similarly, heat treatment negatively impacted the anti-carcinogenetic and anti-bacterial capacities of garlic [16]. Additionally, boiling treatment reduced the fibril degradation ability yet not the anti-amyloidogenic activity of garlic [18]. In sum, alliin-derived OSCs contribute to the anti-neuroinflammatory capacity of raw garlic.

### 2.3. Anti-Neuroinflammatory Activities of OSCs

#### 2.3.1. Inhibitory Effect of OSCs on NO Production and iNOS Gene Expression

**Figure 4:**
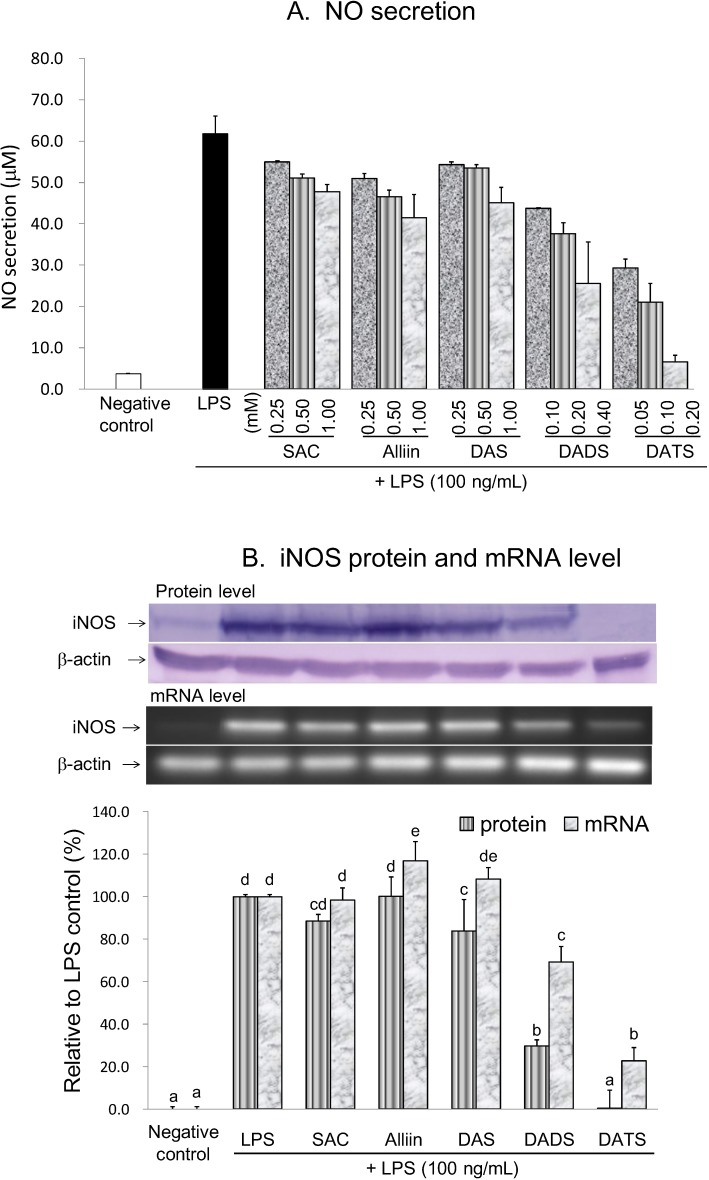
Effects of the selected OSCs on the NO production (**A**); iNOS protein and mRNA levels (**B**) in LPS-activated BV2 microglia. In part B, the treatment concentration is 1000 µM for SAC, alliin and DAS; 400 µM for DADS; and 200 µM for DATS. The values are expressed as means ± SD of triplicate tests. Means not sharing a common letter were significantly different (*p* < 0.05) when analyzed by ANOVA and Duncan’s multiple range test.

This study also evaluated the anti-neuroinflammatory capacities of five OSCs, *i.e.*, SAC, alliin, DAS, DADS, and DATS. Alliin, SAC and DAS at concentrations of ≤1 mM, DADS at concentrations of ≤400 µM, and DATS at concentrations of ≤200 µM did not affect cell viability. Thus, the anti-neuroinflammatory activities were evaluated using concentrations lower than those of their respective cytotoxic dosage. Analysis results indicated that SAC, alliin and DAS inhibited LPS-induced NO production in a dose-dependent manner at concentrations ranging from 250 to 1000 µM (Figure 4). At a concentration of 1000 µM, SAC, alliin and DAS inhibited 24.2%, 35.1%, and 28.9%, respectively, of LPS-induced NO production. In contrast, DADS and DATS exerted an obvious dose-dependent inhibitory effect at concentrations of 100–400 µM and 50–200 µM, respectively. At the highest tested concentration, DADS and DATS attenuated 62.4% and 97.4%, respectively, of the LPS-induced NO production. Furthermore, in order to investigate easily the involved regulating mechanism, we selected the most effective concentration of each individual compound to undertake the subsequent experiments. At a concentration of 1000 µM, SAC and alliin did not diminish the LPS-induced iNOS protein and mRNA expression. DAS (1000 µM) slightly attenuated LPS-induced protein expression, yet not mRNA level of iNOS. Nevertheless, 400 µM DADS and 200 µM DATS significantly decreased both iNOS protein and mRNA levels in LPS-treated BV2 microglia. Obviously, the inhibitory capacity of the five tested OSCs on NO production was ranked as DATS > DADS > alliin, SAC and DAS (Table 2).

**Table 2:** Inhibitory rate of the selected OSCs on the proinflammatory mediator secretion and mRNA expression in LPS-activated RAW 264.7 macrophages.

	Negative Control	LPS	SAC	Alliin	DAS	DADS	DATS
			1 mM	1 mM	1 mM	0.40 mM	0.20 mM
Inhibition of NO secretion (%)	100.0	0.0	24.2	35.1	28.9	62.4	94.9
Inhibition of iNOS protein (%)	100.0	0.0	11.6	−0.2	16.2	70.2	99.7
Inhibition of iNOS mRNA (%)	100.0	0.0	1.6	−16.9	−8.3	30.8	77.4
Inhibition of TNF-α secretion (%)	100.0	0.0	10.1	19.9	25.6	42.4	67.4
Inhibition of IL-1β secretion (%)	100.0	0.0	30.3	27.1	19.1	44.0	69.5
Inhibition of MCP-1 secretion (%)	100.0	0.0	18.4	10.0	14.7	51.0	85.1
Inhibition of TNF-α mRNA (%)	100.0	0.0	16.2	−11.9	9.0	43.6	79.5
Inhibition of IL-1β mRNA (%)	100.0	0.0	5.1	−13.3	−1.0	37.9	53.2
Inhibition of MCP-1 mRNA (%)	100.0	0.0	3.5	−5.7	14.5	33.5	81.6

#### 2.3.2. Inhibitory Effect of OSCs on TNF-α, IL-1β, and MCP-1 Secretion and Gene Expression

**Figure 5:**
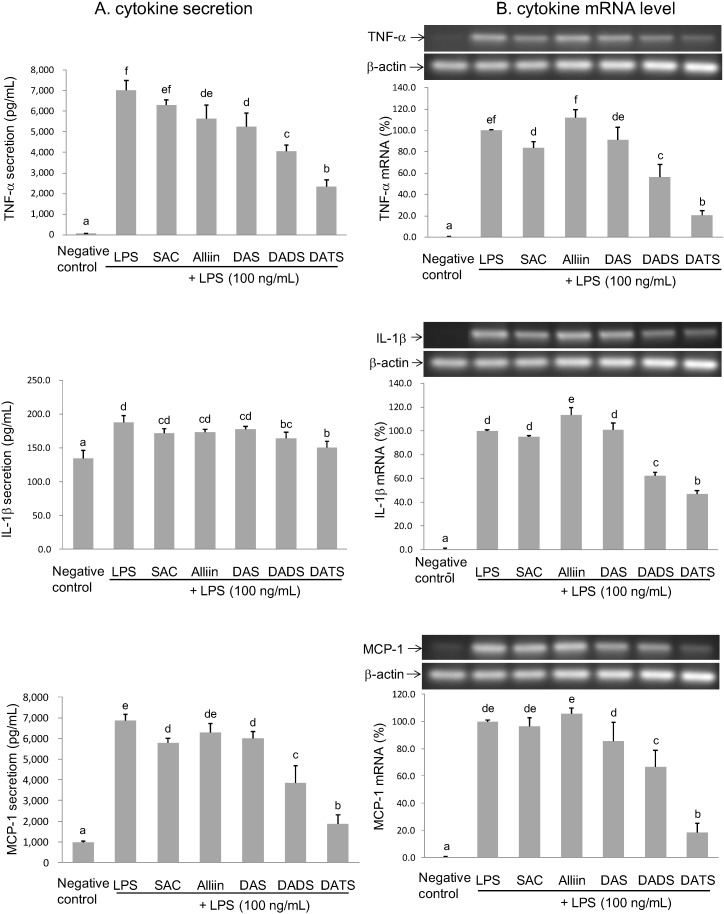
Effects of the selected OSCs on TNF-α, IL-1β and MCP-1 secretion (**A**) and mRNA level (**B**) in LPS-activated BV2 microglia. The treatment concentration is 1000 µM for SAC, alliin and DAS; 400 µM for DADS; and 200 µM for DATS. The values are expressed as means ± SD of triplicate tests. Means not sharing a common letter are significantly different (*p* < 0.05) when analyzed by ANOVA and Duncan’s multiple range test.

Figure 5 and Table 2 shows the extent to which OSCs affect proinflammatory cytokine production in LPS-activated BV2 microglia. At a concentration of 200 µM, DATS could inhibit >50% of LPS-induced TNF-α, IL-1β, and MCP-1 secretions (67.4%, 69.5% and 85.1%), which was the most potent suppressor. DADS at a concentration of 400 µM decreased 42.4%, 44.0% and 51.0%, respectively, of LPS-induced TNF-α, IL-1β, and MCP-1 secretions, which was the moderate suppressor. In contrast, 1000 µM SAC, alliin and DAS only slightly attenuated TNF-α secretion (10.1%, 19.9% and 25.6%, respectively), yet did not affect LPS-induced IL-1β and MCP-1 secretion, which were poor suppressors. Many *Allium* OSCs including SAC, allicin, DADS, DATS, and ajoene, possess an anti-inflammatory capacity [12,13,14]. Correspondingly, this study compared the anti-neuroinflammatory capacities of five OSCs, based on their abundance in different garlic preparations, to more thoroughly elucidate the active anti-neuroinflammatory compounds of garlic. According to those results, alliin, SAC and DAS did not attenuate proinflammatory cytokine secretion in LPS-activated BV2 microglial cells at concentrations up to 1 mM and, therefore, were inadequate in terms of anti-neuroinflammation. Conversely, DATS and DADS significantly inhibited LPS-induced pro-inflammatory cytokine secretion and expression at a concentration of 200 µM and 400 µM, respectively. Of the five tested OSCs, DATS was the most potent anti-neuroinflammatory agent. Apparently, more tethered sulfur atoms imply a greater anti-neuroinflammatory capacity of OSCs. Zhang and Parkin indicated recently that S-alk(en)ylmercaptocysteine but not the corresponding monosulfide species, S-alk(en)yl cysteine, could inhibit NO production in LPS-activated RAW 264.7 macrophages. Correspondingly, they concluded that the disulfide bond is an essential structural moiety for the NO-suppressing activity of *Allium* thiosulfinates [19]. Although not thiosulfinates, SAC, alliin and DAS lack a disulfide bond and might be related to their inadequate anti-neuroinflammatory capacity. Moreover, according to our results, increasing the number of tethering sulfur atoms facilitated the anti-neuroinflammatory capacity of diallyl sulfides. Related studies have observed a similar structure-activity correlation for the anti-inflammatory capacity evaluated by LPS-activated RAW 264.7 macrophages, apoptosis inducing capacity in human prostate cancer cells, suppressing effect on oxidized LDL-stimulated vascular cell adhesion molecules expression in endothelial cells, inhibiting activity on migration and invasion of human colon cancer cells, and hepatic phase 2 detoxification enzyme inducing capacity [14,20,21,22,23]. In sum, the number of tethering sulfur atoms appears to be an important determinant for the anti-neuroinflammatory capacity of OSCs.

### 2.4. Inhibitory Effect of Garlic Extracts and OSCs on NF-κB Binding Activity

**Figure 6:**
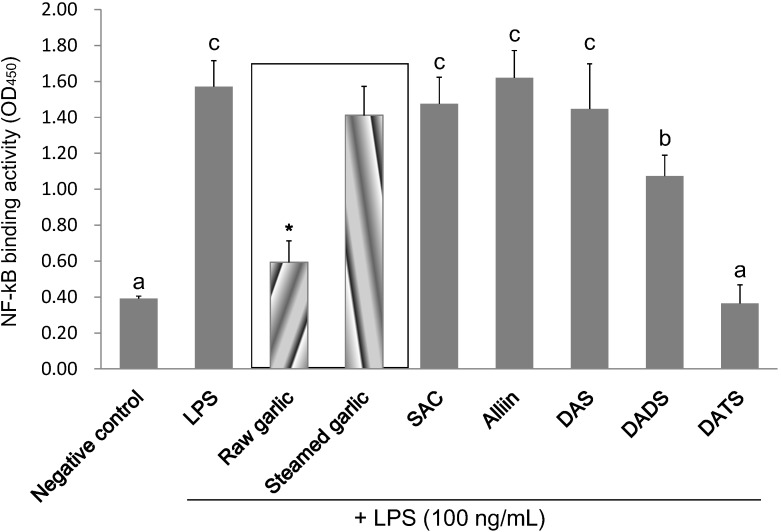
Effects of garlic extracts and the selected OSCs on the NF-κB binding activity in LPS-activated BV2 microglia. The treatment concentration is 5 mg/mL for garlic extracts; 1000 µM for SAC, alliin and DAS; 400 µM for DADS; and 200 µM for DATS. The values are expressed as means ± SD of triplicate tests. Means not sharing a common letter were significantly different (*p* < 0.05) when analyzed by ANOVA and Duncan’s multiple range test. * indicates that tested sample is significantly different from LPS (*p* < 0.05).

Owing to that NF-κB is the convergent transcription factor of inflammatory gene expression, this study determined the DNA binding activity of nuclear p65 by using a commercial ELISA-based kit. According to Figure 6 and Table 3, LPS treatment markedly increased the NF-κB binding activity of BV2 cells. At a concentration of 5 mg/mL, raw garlic extract significantly suppressed 82.9% of NF-κB activation in LPS-activated BV2 microglial cells. In contrast, steamed garlic extract did not inhibit NF-κB activation. Similarly, as expected, 200 µM DATS completely suppressed NF-κB activation, which was the most potent inhibitor among the five tested OSCs. DADS (400 µM) also significantly inhibited LPS-induced NF-κB activation (42.2%). Conversely, 1000 µM of SAC, alliin and DAS did not attenuate NF-κB activation. Inhibiting NF-κB activation appeared to be a molecular mechanism responsible for the anti-neuroinflammatory capacity of raw garlic extract, DATS and DADS.

**Table 3:** Inhibitory rate of garlic extracts and selected OSCs on the NF-κB binding activity in LPS-activated RAW 264.7 macrophages.

	Negative Control	LPS	Raw Garlic	Steamed Garlic	SAC	Alliin	DAS	DADS	DATS
			5 mg/mL	5 mg/mL	1 mM	1 mM	1 mM	0.4 mM	0.2 mM
Inhibition of NF-κB activation (%)	100.0	0.0	82.9	13.6	8.0	−4.1	10.6	42.2	102.4

According to a related investigation, blocking LPS-induced dimerization of toll-like receptor-4 and subsequent NF-κB activation acts as the underlying mechanism of the anti-inflammatory activity of garlic [24]. A recent study indicated that DADS inhibits LPS-induced microglia activation and neuroinflammation by suppressing NF-κB activation and upstream Akt, as well as mitogen-activated protein kinase signaling pathway [25]. Despite the identification of the molecular mechanism, especially those regarding affected intracellular signaling pathways and key transcriptional factors, underlying bioactivities of diallyl polysulfides, the initial acting targets still remain unclear. An increasing amount of recent evidence has indicated that OSCs should directly react with sulfhydryl groups of vital biological molecules to exert their biofunction [26]. Indeed, the reactivity of diallyl sulfides with reduced glutathione was correlated with their phase 2 enzyme inducing potency [27]. Benavides *et al.* found that diallyl polysulfides react with glutathione via nucleophilic substitution to liberate H_2_S, a cardioprotective vascular cell signaling molecule, thereby contributing to the vasoactivity of garlic [26]. S-Alk(en)ylmercaptocysteine could also react with intracellular glutathione to indirectly activate the redox signaling pathway [19]. However, according to Hosono *et al.* DATS exerted its apoptotic activity on colon cancer cells through direct oxidative modification on cysteine residue of β-tubulin [28]. Based on the same oxidative modification, DATS interacted directly with cysteine 288 residue of Kelch-like ECH-associated protein-1 (Keap1), *i.e.*, a cytosolic repressor of nuclear factor-erythroid-2-related factor-2 (Nrf2), thereby inducing the Nrf2 activation and upregulated phase 2 detoxification enzyme expression [29]. A recent study demonstrated that a cyclohexene derivative, TAK-242, inhibits TLR4 signalling by binding directly to cysteine 747 residue of TLR4 and has potent therapeutic effects in an *E. coli*-induced mouse sepsis model [30]. Based on the above developments, we can infer that dially polysulfides might directly attack the cysteine residue of TLR-4 and, therefore, block LPS-induced TLR-4 dimerization and inhibit microglial activation. However, this inference warrants further investigations for verification.

In addition to anti-neuroinflammation, garlic and OSCs targeted multiple pathways to alleviate neurodegenerative disease and the therapeutic effects have been evaluated in various models of neurodegenerative diseases. For example, crude garlic extract could improve the learning and memory of scopolamine-induced amnesia mice [31]. Aged garlic extract, SAC and DADS ameliorated Aβ-induced neurotoxicity and cognitive impairment in Alzheimer’s transgenic model Tg2576 [32]. DATS treatment improved motor performance and ameliorated pathological changes in ALS transgenic mouse model [33]. However, according to pharmacokinetic studies, the garlic diallyl sulfides were rapidly metabolized into sulfate in the liver and could not be detected in the circulation, explaining why the role of these diallyl sulfides in functioning as *in vivo* active components of garlic is a contentious issue. In contrast, the highly bioavailable SAC is considered as the active components of garlic [34]. Interestingly, the ethyl acetate fraction (not the water fraction) of aged garlic extract was found recently to have the highest antioxidant activity and could ameliorate Aβ-induced neurotoxicity and cognitive impairment in Tg mice. Furthermore, various sulfides such as dimethyl disulfide were identified as the most abundant compounds in the ethyl acetate fraction. Above results imply that the neuroprotective effect of aged garlic extract against Alzheimer’s disease was collaboratively attributed to various sulfides [35].

## 3. Experimental Section

### 3.1. Chemicals

Four OSCs (*i.e.*, alliin, SAC, DAS, and DADS) were purchased from Sigma-Aldrich Co (St. Louis, MO, USA). DATS was obtained from the ChromaDex (Irvine, CA, USA). LPS (*Escherichia coli* O26:B6), 3-(4,5-dimethyldiazol-2-yl)-2,5-diphenytetrazolium bromide (MTT), sulphanilamide, naphthylethylenediamine, and nitro blue tetrazolium chloride/5-bromo-4-chloro-3-indolyl phosphate p-toluidine salt (NBT/BCIP) tablets were purchased from Sigma-Aldrich. All other chemicals and solvents used were of reagent analytical grade.

### 3.2. Preparation of Raw- and Steamed-Garlic Ethanolic Extracts

Raw garlic cloves were purchased from a local supermarket (Hsinchu, Taiwan), and a portion of them were steamed with boiling water for 10 min. After peeling, 160 g of raw- and steamed-garlic cloves were ground into purees and left standing for 10 min. Next, 320 mL of 90% ethanol was added, and the suspension was shaken at 150 rpm at room temperature for 2 h. Following centrifugation at 10,000× *g* for 30 min, the supernatant was vacuum-dried at ambient temperature to remove the ethanol. Finally, the extract solids were suspended in phosphate-buffered saline (PBS) to a final concentration of 0.5 g/mL.

### 3.3. HPLC and LC-MS Analyses

HPLC analysis was performed with a Hitachi Primaide 1110 pump and 1430 diode-array detector (Hitachi, Milford, MA, USA). Compounds were separated on a 150 mm × 3 mm i.d. × 3 µm particle C18 Hypurity column (Thermo Hypersil Division, Keystone, Bellefonte, PA, USA), at 38 °C with a controlled flow rate of 0.4 mL/min and set wavelength of 208 nm. Gradient elution program for the two-solvent system (solvent A, 0.1% formic acid; solvent B, acetonitrile) was as follows: 0 min, 0% B; 3 min, 10% B; 5 min, 15% B; 25 min, 27% B; 35 min, 50% B; 42 min, 80% B; 46 min, 90% B; and hold at 90% B for 10 min. Injection volume was 10 µL. LC-MS analyses were performed in the Instrument Center of Chiao Tung University (Hsinchu, Taiwan). Electrospray ionization (ESI) mass spectra were obtained with a Micromass Quattro triple quadrupole mass spectrometer link to an electrospray source operated in positive ion mode. The acquisition parameters were as follows: drying N_2_ temperature of 250 °C; flow rate of 400 mL/h; capillary current of 3.0 kV; capillary exit RF amplitude of 0.3 V; and mass range measured *m*/*z* of 100–1000.

### 3.4. Cell Culture Experiment

BV2 microglial cells were cultured in a DMEM medium containing 10% fetal bovine serum (Invitrogen, Carlsbad, CA, USA). The effect of garlic extracts and OSCs on the proinflammatory mediator secretion was examined by seeding BV-2 cells into 48-well plates at a density of 2 × 10^5^ cells/well for 24 h. After washing with PBS, the cells were exposed to LPS (100 ng/mL) in the presence of garlic extract (1.25–5.00 mg/mL) or OSCs (indicated concentration) for 24 h. The conditioned medium was collected for pro-inflammatory mediator determination and, then, the cells were further incubated in medium containing 0.5 mg/mL MTT for 3 h. Following removal of the supernatant, formation of formazan was measured at 540 nm using a microplate reader to evaluate cell viability.

### 3.5. Determination of Amounts of Secreted NO, TNF-α, IL-1β, and MCP-1

The concentrations of NO in the culture medium were determined as nitrite, a major stable product of NO, by Griess reagent (1% sulphanilamide and 0.1% naphthylethylenediamine in 5% H_3_PO_3_). The nitrite concentrations were calculated based on a standard curve generated by known concentrations (5–100 μM) of sodium nitrite. The TNF-α, IL-1β (eBioscience, San Diego, CA, USA) and MCP-1 (BioLegend, San Diego, CA, USA) levels of the conditioned medium were measured by the ELISA kits based on the manufacturer’s instructions.

### 3.6. Evaluation of iNOS Protein Levels

BV-2 cells were seeded in a 6-cm culture dish and exposed to LPS (100 ng/mL) with garlic extract (5 mg/mL) or OSCs (indicated concentration) for 12 h. Protein samples from the cell lysate were separated by 8% SDS-PAGE and then transferred to a polyvinylidene fluoride membrane. The blotted membrane was incubated with anti-iNOS (Cayman Chemical, Ann Arbor, MI, USA) and anti-β-actin antibodies (Biovision, Mountain View, CA, USA), respectively, overnight at 4 °C. After further incubation with alkaline phosphatase-conjugated secondary antibody for 1 h, protein bands were visualized by reacting with a NBT/BCIP solution. Finally, the bands of iNOS protein were quantified by a software-supported photo-imager (ImageMaster VDS; Amersham Pharmacia Biotech Co., Piscataway, NJ, USA) and were normalized with β-actin.

### 3.7. Evaluation of iNOS, TNF-α, IL-1β, and MCP-1 mRNA Levels

Cells were collected after their incubation with garlic extract (5 mg/mL) or OSCs (indicated concentration) in the presence of 100 ng/mL LPS for 6 h. The iNOS, TNF-α, IL-1β, and MCP-1 mRNA levels were then determined using a semi-quantitative reverse transcription polymerase chain reaction (RT-PCR). Total RNA was first isolated using Trizol reagent (Invitrogen), reverse-transcribed to cDNA, and then amplified with PCR using a commercially available kit (Promega, Madison, WI, USA). Additionally, 20 µL aliquots of each PCR sample were subjected to electrophoresis in 2% agarose gel, stained with ethidium bromide, and visualized under an ultraviolet light. Moreover, band intensities of the PCR products were quantified using a software-supported photo-imager and normalized with β-actin. Sequences of the PCR primers were as follows: 5′-CAGTT CTGCGCCTTTGCTCAT-3′ (forward) and 5′-GGTGGTGCGGCTGGACTTT-3′ (backward) for iNOS; 5′-TTCTGTCCCTTTCACTCACTGG-3′ (forward) and 5′-TTGGTGGTTTGCTACGACGTGG-3′ (backward) for TNF-α; 5′-ATGGCAATCGTTCCTGAACTCAAC-3′ (forward) and 5′-CAGGACAGG TATAGATTCTTTCCTTT-3′ (backward) for IL-1β; 5′- TTCCTTCTTGGGGTCA-GCACAGAC-3′ (forward) and 5′-ACTGAAGCCAGCTCTCTCTTCCTC-3′ (backward) for MCP-1; and 5′-AGGC CCAGAGCAAGAGAG-3′ (forward) and 5′-GGGTGTTGAAGGTCTCAAAC-3′ (backward) for β-actin.

### 3.8. Evaluation of NF-κB Binding Activity

The nuclear p65 DNA binding activity was evaluated by using a commercial kit (Cayman Chemical). Briefly, cellular nuclear proteins were extracted following incubation of the cells with garlic extract (5 mg/mL) or OSCs (indicated concentration) in the presence of 100 ng/mL LPS for 1 h. Nuclear extracts (5 µg protein) were added to the wells of a plate pre-coated with NF-κB response element DNA. After incubation overnight at 4 °C, the bound p65 was detected sequentially by a specific NF-κB p65 antibody and a horseradish peroxidase-conjugated secondary antibody. Finally, the absorbance at 450 nm was measured.

### 3.9. Statistical Analysis

Analysis results are expressed as mean ± standard deviation from at least three independent tests. The significance of group differences was examined using one-way ANOVA, and the Duncan’s multiple range test was conducted to make multiple comparisons. Differences were considered significant at *p* < 0.05. All statistical analyses were performed with SPSS software version 17.0 (SPSS, Inc., Chicago, IL, USA).

## 4. Conclusions

As expected, raw garlic extract inhibited NO, proinflammatory cytokine, and chemokine production by through suppression of NF-κB activation in LPS-activated BV2 microglia; it also had a potent anti-neuroinflammatory capacity. Additionally, steaming pretreatment abolished both the anti-neuroinflammatory capacity and alliin-derived OSCs formation of garlic simultaneously. In sum, this study demonstrates that alliinase catalysis and chemical transformation are essential for the formation of active OSCs, which are responsible for the anti-neuroinflammatory capacity of garlic. Based on above, it is suggested that consumers to crush or cut raw garlic before cooking in order to obtain more health benefits of garlic. As one of the most potent anti-neuroinflammatory components of garlic, DATS is highly promising for use as a dietary agent to prevent inflammation-related neurodegenerative disease.
